# Identification of Spt5 Target Genes in Zebrafish Development Reveals Its Dual Activity *In Vivo*

**DOI:** 10.1371/journal.pone.0003621

**Published:** 2008-11-03

**Authors:** Keerthi Krishnan, Nathan Salomonis, Su Guo

**Affiliations:** 1 Department of Biopharmaceutical Sciences, Programs in Human Genetics and Pharmaceutical Sciences and Pharmacogenomics, University of California San Francisco, San Francisco, California, United States of America; 2 Gladstone Institute of Cardiovascular Disease, San Francisco, California, United States of America; Institute of Genetics and Molecular and Cellular Biology, France

## Abstract

Spt5 is a conserved essential protein that represses or stimulates transcription elongation *in vitro*. Immunolocalization studies on Drosophila polytene chromosomes suggest that Spt5 is associated with many loci throughout the genome. However, little is known about the prevalence and identity of Spt5 target genes *in vivo* during development. Here, we identify direct target genes of Spt5 using *fog^sk8^* zebrafish mutant, which disrupts the *foggy/spt5* gene. We identified that *fog^sk8^* and their wildtype siblings differentially express less than 5% of genes examined. These genes participate in diverse biological processes from stress response to cell fate specification. Up-regulated genes exhibit shorter overall gene length compared to all genes examined. Through chromatin immunoprecipitation in zebrafish embryos, we identified a subset of developmentally critical genes that are bound by both Spt5 and RNA polymerase II. The protein occupancy patterns on these genes are characteristic of both repressive and stimulatory elongation regulation. Together our findings establish Spt5 as a dual regulator of transcription elongation *in vivo* and identify a small but diverse set of target genes critically dependent on Spt5 during development.

## Introduction

The production of functional mRNA involves multiple steps that include transcription initiation, elongation, and termination. The initiation step is thought to be the major regulatory point for most genes *in vivo*, despite that promoter-proximal stalled RNA polymerase II (RNAPII) has been detected at several loci including *hsp70* [1], *HIVLTR* [2], *c-myc* [3], and *c-fos* [4]. Recently, it has been observed that promoters of many genes are poised for activation, as assessed by chromatin status and RNAPII occupancy in human Hela cells, mammalian embryonic stem cells, and *Drosophila* embryos [5]–[9]. These observations suggest that regulation at the level of transcription elongation may be more prevalent than previously thought. However, the distinct role of individual transcription elongation factors in critically regulating elongation of target genes remains unclear.

In recent years, biochemical studies have identified over a dozen proteins and a small nuclear RNA, which regulate RNAPII elongation *in vitro* [10]–[17]. Among these molecules, Spt5, an evolutionarily conserved protein, is the focus of this study. Spt5, together with Spt4 and Spt6, was initially discovered by genetic studies in *Saccharomyces cerevisiae* [18], and later found to be essential for transcription via modulating chromatin structure [19], [20]. A search for factors that inhibit transcription elongation in the presence of the nucleotide analog 5,6-dichloro-1-β-D-ribofuranosylbenzimidazole (DRB) led to the biochemical purification of Spt4 and Spt5 as a tight complex named DSIF (DRB Sensitivity Inducing Factor), which has both repressive and stimulatory activity on transcription elongation *in vitro* [21]. Spt5 has also been identified independently as a protein required for HIV-1 Tat trans-activation in fractionated extracts [22]. In addition, Spt5 interacts and modulates activity of factors implicated in mRNA maturation and surveillance [23]–[25].

At the phenotypic level, the *in vivo* role of Spt5 in multi-cellular organisms has been revealed through genetic analyses in *C. elegans*, *Drosophila*, and zebrafish. Inactivation of Spt5 through RNAi in *C. elegans* arrest embryonic development prior to gastrulation [26]. In *Drosophila*, a hypomorphic mutation in *spt5* displays defects in the expression of segmental patterning genes [27]. At the molecular level, Spt5 localization on polytene chromosomes and chromatin immunoprecipitation studies reveal broad distribution of Spt5 at sites of active transcription as well as at the un-induced heat shock gene promoters [28]–[30]. In zebrafish, whereas a hypomorphic mutation in *spt5* (the *fog^m806^* allele) displays distinct defects in neuronal development [31], severe truncation (the *fog^sk8^* allele) or deletion (the *fog^s30^* allele) of the gene product causes broad deficits in embryonic development [32]. Together, these analyses establish functional significance of Spt5 *in vivo*, implicate intricate regulatory mechanisms that remain to be elucidated, and suggest that Spt5 may regulate a large number of target genes *in vivo*. Despite these insights, only the heat shock protein-encoding *hsp70* and HIV viral genes, have been demonstrated to be regulated at the level of transcription elongation by Spt5 in intact cells [29], [33], whereas induction of the immediate early gene *c-fos* appears to depend on the stimulatory activity of Spt5 [34]. Thus, an important question remains: What other genes, if any, are critically dependent on Spt5 *in vivo*?

Here we report expression profiling of over 10,000 protein-coding genes using ∼24 hours post fertilization (hpf) zebrafish embryos. Our results identified a surprisingly small fraction (<5%) of genes that were differentially expressed between *fog^sk8^* mutants and their wildtype (WT) siblings. Pathway analyses revealed that these differentially expressed genes are involved in diverse biological pathways that range from stress response to cell fate specification. A gene structure analysis showed that genes upregulated in *fog^sk8^* embryos had a significantly shorter length, compared to the average gene length in zebrafish. Furthermore, *in vivo* chromatin immunoprecipitation (ChIP) on selected set of differentially expressed genes in 24 hpf WT embryos uncovered Spt5 target genes: among the target genes repressed by Spt5 (including *gadd45b*, *c-fos*, *hsp70*, *smoothened*, and *follistatin*), RNAPII occupancy was higher at the 5′ than 3′ end of these genes; among the target genes activated by Spt5 (including *lunatic fringe* and *tropomyosin a*), RNAPII occupancy was detected at both their 5′ and 3′ ends. ChIP analyses in *fog^sk8^* mutant embryos identified *gadd45b* that exhibited significantly increased RNAPII occupancy at the 3′ end compared to that in WT embryos, whereas other target genes unexpectedly showed reduced RNAPII occupancy at both 5′ and 3′ ends. This observation highlights the dynamic and complex nature of elongation regulation *in vivo*. Taken together, our results establish Spt5 as a dual regulator of transcription elongation *in vivo* and reveal a small but diverse set of direct target genes.

## Results

### Spt5 protein is significantly reduced in 24 hpf *fog^sk8^* mutant embryos

**Figure 1:**
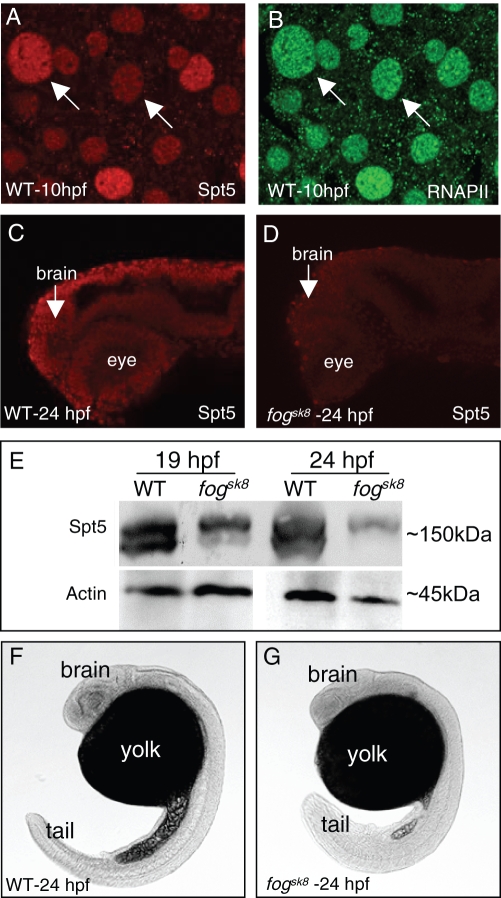
Spt5 protein is significantly decreased in *fog^sk8^* embryos. (A–B) Whole mount immunofluorescence detection of Spt5 and RNAPII in zebrafish embryos, showing the nuclear localization pattern of Spt5 protein. (C–D) Whole mount immunofluorescence detection of Spt5 in WT and *fog^sk8^* embryos at 24 hpf, showing overall decrease of Spt5 protein in *fog^sk8^* embryos. Confocal images of lateral views are shown, with the anterior to the left and dorsal up. (E) Western blot of wild-type (WT) and *fog^sk8^* embryonic extracts at 19 hpf and 24 hpf shows substantial decrease in maternal Spt5 protein in the mutant. (F–G) Light microscopic view of WT and *sk8* embryos at 24 hpf, showing the gross morphological phenotype of the mutant embryos.

The *foggy/spt5* transcript is broadly distributed throughout the developing embryo [31], [32]. To gain insights into the level and distribution of Spt5 protein, an antibody directed against the C-terminal end of the zebrafish Spt5 protein was generated. Double-labeling with 8WG16, an antibody that recognizes RNAPII, demonstrated prominent nuclear distribution of Spt5 (Fig. 1A–B). The *fog^sk8^* mutation (Fig. 1F–G) is predicted to yield a non-functional zygotic gene product, and the maternal transcript is undetectable after 10 hours post fertilization (hpf) [32]. However, the status of the maternal protein was unclear. The *sk8* mutant phenotype is first visible around 19 hpf, suggesting the presence of maternal Foggy protein at earlier stages of development. Immunostaining (Fig. 1C–D) showed that maternal Spt5 protein was significantly reduced in *fog^sk8^* mutant embryos as compared to WT siblings. Western blotting (Fig. 1E) detected two bands around the predicted size of zebrafish Spt5 in WT embryos. Since Spt5 is known to be phosphorylated [14], [34]–[38] and methylated [39], these bands may represent forms of Spt5 with different post-translational modifications. Although the intensity of both bands was reduced in the *fog^sk8^* mutant, the faster migrating form appeared more severely affected (Figure 1E). This result indicates that *fog^sk8^* mutant at 24 hpf exhibits a significant reduction but not complete absence of Spt5 protein.

### Expression profiling reveals a small number of differentially expressed genes in 24 hpf *fog^sk8^* embryos

**Figure 2:**
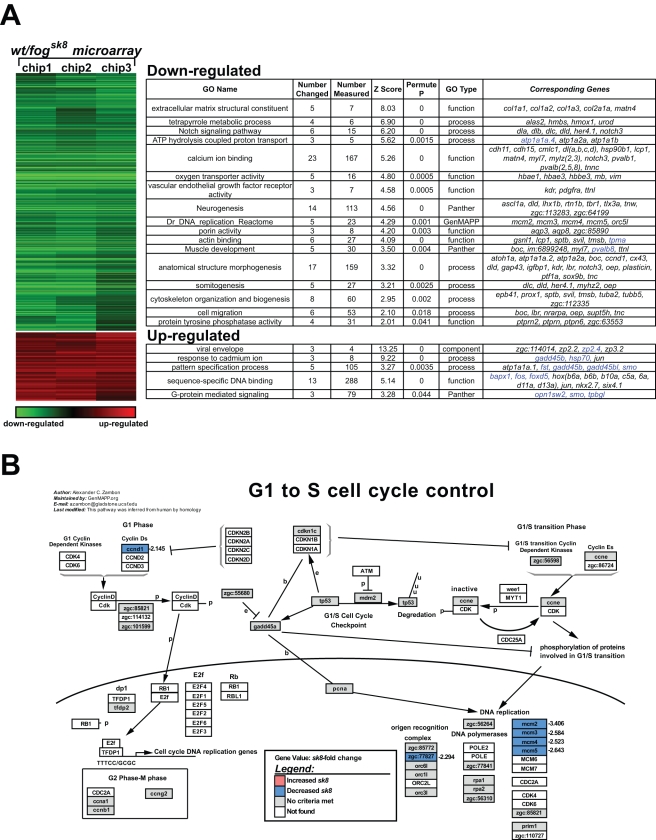
Gene expression profiling reveals that the expression of a small percentage of genes is affected in *fog^sk8^* mutant embryos. (A) HOPACH cluster diagram of the microarray data are shown for individual replicates compared with the mean for 24 hpf embryos. 615 probe sets with fold change >2 and t test *P*-value <0.05 are displayed along with Gene Ontology terms and pathways over-represented as determined by a Fisher's exact test permutation based *P*-value. The intensity of down-regulation (green) and up-regulation (red) reflects the magnitude of fold change. Representative genes within the GO categories are shown in the last column. Genes subjected to further analyses in this study are represented in blue color in the last column. (B) G1 to S Cell Cycle pathway is shown as an example of GenMAPP pathway analysis. The pathway map shows genes that are regulated as a group (e.g. *mcm* genes) and genes that are not differentially expressed in *fog^sk8^* embryos but are present on the array (grey color).

To identify potential downstream target genes of Spt5, we performed microarray analysis by hybridizing zebrafish Affymetrix oligoarrays, which contain over 10,000 protein-coding genes, with biotinylated cRNA probes from 24 hpf *fog^sk8^* mutant and WT sibling embryos. 75% of the probe sets displayed above-background signal-to-noise ratio (as determined by microarray absent-present calls), suggesting that a majority of genes on the array have detectable expression at this stage. Interestingly however, only 4.5% of the genes were differentially expressed (absolute fold greater than 2, student t-test *P*<0.05) between *fog^sk8^* and WT siblings. The 2-fold cutoff was used because an expression change of less than 2 fold was often not validated by quantitative PCR analysis (data not shown). Among the differentially expressed genes, 97 (∼1% of the total genes examined) were up-regulated (ranging from +2- to +10-fold), and 358 (∼3.5% of the total genes examined) were down-regulated (from −2- to −300-fold) in *fog^sk8^* embryos (Fig. 2A). Many of the differentially expressed genes have previously been shown to display dynamic spatial and temporal expression patterns [40]. Four genes (*gch*, *pvalb*, *hoxb6*, *dlx2*) were validated through whole mount *in situ* analysis (Supplemental Fig. S1) and 28 other genes were further validated through quantitative RT-PCR analysis (Supplemental Tables S1 and S2).

To identify biological pathways that might be regulated by Spt5, we employed GenMAPP [41] and associated tools [42] (http://www.genmapp.org/go_elite) to analyze pathway and gene ontology over-representation in our differentially expressed gene dataset. This analysis found that genes induced by environmental or cellular stress (such as *gadd45b* and *hsp70*) or encoding certain transcription factors (such as *foxd5* and *fos*) were upregulated, whereas genes that are constituents of the extracellular matrix (e.g. *collagen*) and sodium/potassium ion transporters (e.g. *atp1a1a*) were down-regulated in *fog^sk8^* embryos (Fig. 2A). The expression of genes previously suggested to be regulated at the level of elongation [10], including *hsp70*, *fos*, *globin*, *tubulin*, and *opsin*, were found altered in *fog^sk8^* embryos. Moreover, the expression of multiple genes in the cell cycle pathway was reduced in *fog^sk8^* embryos (Fig. 2B). In contrast, genes from major developmental pathways (e.g. fibroblast growth factor, wingless), although present on the array, were largely unaffected in *fog^sk8^* embryos (Supplementary Table S3). In addition, RNA processing genes and protein biosynthesis genes (ribosome constituents) were under-represented in our differentially expressed gene dataset (data not shown). It is worth pointing out that many differentially expressed were novel, and were thus not assignable to specific GenMAPP pathways. Taken together, these results indicate that the lack of zygotic Spt5 protein only affects the expression of a small percentage of genes at 24 hpf, and these genes are involved in a number of distinct biological pathways.

### Genes that are upregulated in *fog^sk8^* embryos are shorter in gene length compared to all genes examined

**Figure 3:**
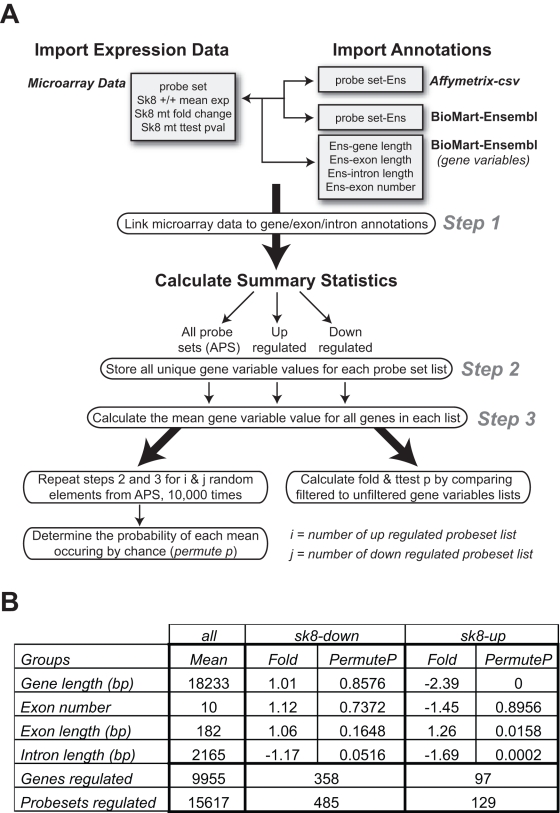
Permutation analysis uncovers a significant shorter length of those genes that are up-regulated in *fog^sk8^* mutant embryos. (A) Algorithm design for assessing global changes in overall gene length. Unique Ensembl genes linked to probesets were obtained from the Affymetrix website and BioMart, and genomic gene, exon and intron length were also obtained from BioMart and summarized for up-, down-regulated and all array probesets (AAPs). Folds relative to AAPs linked genes were calculated as well as permutation based p-values by randomly selecting input probeset lists of the same size as the regulated sets from AAPs, over 10,000 iterations. (B) Table summarizing the permutation based analysis of the structural features of genes differentially expressed in *fog^sk8^* embryos. Up-regulated genes have significantly shorter gene length, shorter intron length and longer exon length than other genes on the array. Down-regulated genes do not show significantly different gene structure requirements compared to other genes.

Little is known as to what signatures may subject a gene to Spt5 regulation. To determine if differentially expressed genes have common features in gene structures, we performed bioinformatics analysis on these gene sets. To assess the extent and overall likelihood of a difference between the length of *fog^sk8^* –dependent genes and that of all other genes in the array, probe sets from the microarray were linked to gene structure data from the Ensembl database, the mean of different gene features (e.g., genomic length) was computed, and fold change and *P* values were calculated via a permutation based analysis (Fig. 3A).

Using this method, we found that genes upregulated in *fog^sk8^* embryos had on average ∼2.39 fold decrease in overall gene length and ∼1.69 fold decrease in intron length, as compared to all gene-linked probe sets on the array (*P*<0.05) (Fig. 3B). These differences were unlikely to occur due to chance, because a fold change larger than or equal to the gene length difference (2.39 fold) was not observed in 10,000 random permutations of all microarray probe sets, and that larger than or equal to the intron length difference (1.69 fold) only occurred twice over 10,000 random permutations (*P* = 0.0002), hence emphasizing the unlikelihood of such events occurring at random. Although this analysis also showed a significant increase in exon length for upregulated genes, the difference was relatively small (1.26 fold). None of the gene structure data differed between downregulated genes and all other genes on the array (Fig. 3B). Taken together, this analysis suggests that genes repressed by Spt5 tend to be shorter in length.

### In vivo chromatin immunoprecipitation (ChIP) reveals Spt5 occupancy patterns on differentially expressed genes in 24 hpf WT embryos

As microarray experiments measure the steady-state levels of mRNA and Spt5 is involved in many aspects of mRNA stability by virtue of its association with many components of the transcriptional machinery , many of the genes differentially expressed in *fog^sk8^* mutants might represent mRNA processing targets of Spt5. In order to better identify genes that are regulated at the elongation level at 24 hpf, we performed chromatin immunoprecipitation (ChIP) assays using antibodies against RNAPII and Foggy.

**Figure 4:**
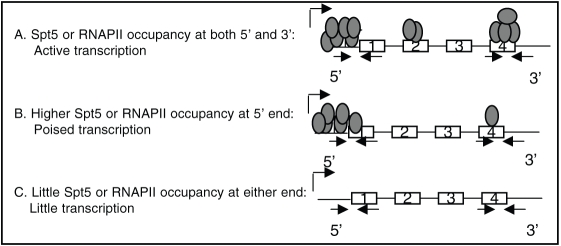
Schemata showing predicted transcriptional status based on Spt5 and RNAPII occupancy.

Based on the current model of regulation at the level of transcription elongation [16], [17], the following patterns of Spt5 and RNAPII occupancy are expected on their target genes (Fig. 4): A) Spt5 target genes that are in a state of productive transcription elongation shall display Spt5 and RNAPII occupancy at both 5′ and 3′ ends of their chromatin. B) Spt5 target genes that are repressed by a promoter-proximal transcription elongation block shall have minimal Spt5 and RNAPII occupancy at 3′ end but significantly more Spt5 and RNAPII occupancy at 5′ end. C) Genes that are not Spt5 targets or are transcriptionally inactive shall lack detectable Spt5 or RNAPII respectively throughout their chromatin.

**Figure 5:**
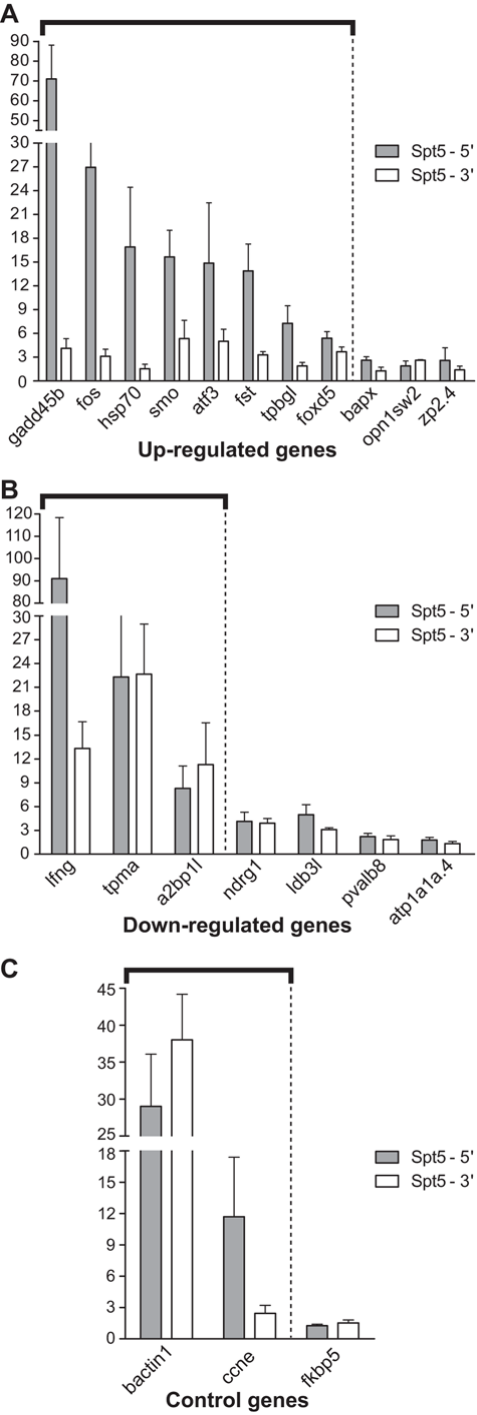
*In vivo* ChIP analysis using Spt5 antibody on differentially expressed genes reveals putative target genes directly bound by Spt5. (A–C) Spt5 occupancy on upregulated, downregulated, and control genes. Y- axis values in the graphs represent fold Foggy occupancy over fold pre-immune serum occupancy levels. Arbitrary cutoff for detectable Foggy fold occupancy over control serum is set at 5. Thick black bars highlight the genes that display significant Spt5 occupancy at either 5′ or 3′ end of the chromatin. The results shown are the average of at least three independent replicates. Error bars are SEM values. Note that the scales are different for each of the graphs.

Based on the significance of expression fold changes, constraints on availability of reliable genomic structure (exon/intron) information, and known gene functionality, 11 upregulated genes, 7 downregulated genes, and 3 control genes were selected for ChIP analysis (Table 1 and Supplementary Table S4) in 24 hpf WT embryos. Among the 11 upregulated genes, 8 (*gadd45b*, *fos*, *hsp70*, *smo*, *atf3*, *fst*, *tpbgl*, *foxd5*) showed high Spt5 occupancy at 5′ end but little at 3′ end, whereas the remaining three genes did not show significant Spt5 occupancy on either end (Fig. 5A and Table 1). Among the 7 downregulated genes, 3 (*lfng*, *tpma*, *a2bp1-like*) showed significant Spt5 occupancy at both 5′ and 3′ ends, whereas the remaining 4 genes did not (Fig. 5B and Table 1). Genes that showed significant Spt5 occupancy are likely to be targets of Spt5, whereas those that did not have significant Spt5 occupancy were either transcriptionally inactive or not directly regulated by Spt5. Two (*beta-actin 1* and *cyclin E*) of the three control genes showed significant Spt5 occupancy (Fig. 5C and Table 1), whose expression was largely unaltered in 24 hpf *fog^sk8^* embryos, suggesting that these transcripts might be regulated by additional elongation factors.

**Table 1:** Summary of Genes Used for *in vivo* CHIP Analysis.

No.	Gene Symbol	Gene Name	GO Terms*	Array/qRT-PCR Fold Change	Genomic length (kb)	Transcript length (kb)	No. of Exons	Degree of Spt5 occupancy	Degree of RNAPII occupancy
**Genes Up-regulated in 24 hpf ** ***fog^sk8^*** ** Mutant Embryos**									
1	*gadd45b*	Growth arrest and DNA-damage-inducible, beta	Stress response	6.34/6.07	1.6	1.2	4	++++	++++
2	*fos*	v-fos FBJ murine osteosarcoma viral oncogene	Transcription Factor	5.68/8.44	2.1	1.7	4	++++	++++
3	*hsp70*	Heat shock protein 70	Stress response	5.6/2.32	3.4	2.3	2	+++	+++
4	*fst*	Follistatin	Pattern specification	2.25/2.07	5.5	3.4	5	+++	++
5	*smo*	Smoothened homolog	Pattern specification	2.02/2.12	5.6	3.0	12	+++	++++
6	*atf3*	Activating transcription factor 3	Transcription Factor	2/1.89	6.1	2.6	4	+++	++
7	*tpbgl*	Trophoblast glycoprotein-like	Receptor	3.24/2.37	2.6	1.7	2	++	+++
8	*foxd5*	Forkhead box D5	Transcription Factor	9.83/10.77	4.1	1.1	2	+	+
9	*bapx*	Bagpipe homeobox 1/ NK3 homeobox 2	Transcription Factor	4.62/4.62	0.5	0.3	2	−	−
10	*zp2.4*	Zona pellucida glycoprotein 2.4	Viral envelope	3.74/6.02	1.8	1.3	8	−	−
11	*opn1sw2*	Opsin 1, short-wave-sensitive 2	Signal transduction	2.72/7.81	2.6	1.3	5	−	−
**Genes Down-regulated in 24 hpf ** ***fog^sk8^*** ** Mutant Embryos**									
1	*lfng*	Lunatic fringe homolog	Signaling	3/3.68	9.4	2.9	8	++++	++++
2	*tpma*	Alpha tropomyosin	Actin binding	2.86/3.41	4.5	1.3	10	+++	+++
3	*a2bp1l*	ataxin 2-binding protein 1-like	Nucleotide binding	10.69/7.83	18.5	1.1	12	++	++
4	*ndrg1*	N-myc downstream regulated gene 1	Cell differentiation	6.04/8.47	19.9	2.3	16	−	+
5	*ldb3l*	LIM domain binding 3 like	Protein binding	3.64/3.44	39.3	1.6	12	−	−
6	*pvalb8*	Parvalbumin 8	Calcium ion binding	17.62/12.23	174.7	0.8	5	−	−
7	*atp1a1a.4*	ATPase, Na+/K+ transporting, alpha 1a.4	Sodium\potassium ATPase	3.37/62.85	7.5	3.2	22	−	−
**Genes Unchanged in 24 hpf ** ***fog^sk8^*** ** Mutant Embryos**									
1	*bactin1*	Beta Actin 1	Cytoskeleton	1.26/−1.8	3.6	1.7	6	+++	++++
2	*ccne*	Cyclin E	Cell cycle	1/1.87	9.8	2.0	12	++	−
3	*fkbp5*	FK506 binding protein 5	Protein folding	1/1.04	9.9	2.4	11	−	−

* GO/MAPP program-suggested gene function.

### ChIP analysis using the 8WG16 antibody in 24 hpf WT embryos reveals RNAPII distribution patterns on the differentially expressed genes

**Figure 6:**
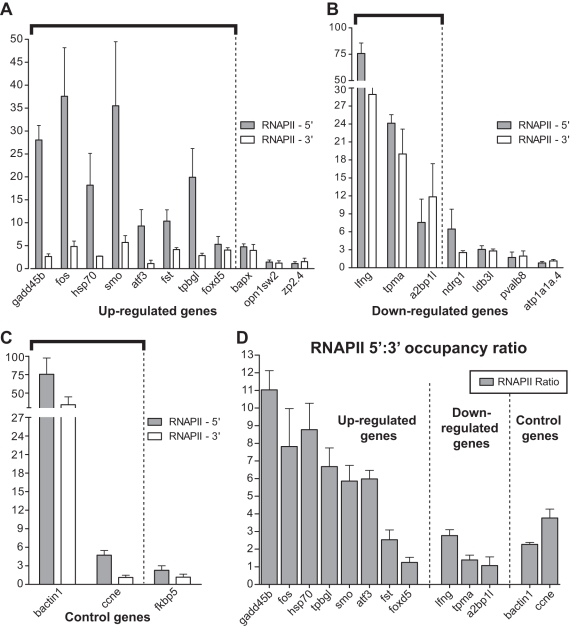
Distinct patterns of RNAPII occupancy at putative Spt5 target genes. (A–D) ChIP data showing RNAPII occupancy in 24 hpf WT embryos on the genes that are up-regulated in *fog^sk8^* embryos (A), genes that are down-regulated in *fog^sk8^* embryos (B), control genes (C), and the ratio of RNAPII occupancy at 5′ vs 3′ end (D). Y-axis values in the graphs represent fold RNAPII occupancy over fold mouse control serum occupancy. Arbitrary cutoff for detectable RNAPII fold occupancy over control serum is set at 5. Thick black bars highlight the genes that display significant RNAPII occupancy at either 5′ or 3′ end of the chromatin. The results shown are the average of at least three independent replicates. Error bars are SEM values.

We next examined RNAPII distribution in 24 hpf WT embryos to determine the transcriptional status of the genes. The 8WG16 antibody was used in our experiments, as it has been successfully used to recognize RNAPII occupancy on the chromatin at both the 5′ and 3′ ends of genes in other species [43]. In general, the RNAPII occupancy pattern correlated well with that of Spt5. Among those that were upregulated in the *fog^sk8^* mutant embryo and also displayed significant Spt5 occupancy (indicating that their expression is repressed by Spt5), more RNAPII was detected at 5′ than 3′ end (Fig. 6A, 6D, and Table 1). This finding is consistent with the idea that the genes repressed by Spt5 are in a poised state of transcription. Among those that were downregulated in the *fog^sk8^* mutant embryo and also displayed significant Spt5 occupancy, significant occupancy of RNAPII was also detected at both 5′ and 3′ ends (Fig. 6B, 6D, and Table 1), indicating that these genes require the stimulatory activity of Spt5 for productive transcription in 24 hpf WT embryos. The genes that exhibited little Spt5 occupancy also showed little RNAPII occupancy, confirming that these genes are not transcriptionally active in 24 hpf WT embryos. Taken together, our results identify *gadd45b*, *fos*, *hsp70*, *tpbgl*, *smo*, *atf3*, *fst*, and *foxd5* as target genes that are repressed by Spt5 at the elongation step, and *lfng*, *tpma*, and *a2bp1-like* as target genes that are activated by Spt5 at the elongation step. Though genes such as fos and hsp70 have been known to be regulated by elongation, our results identify multiple critical developmental regulators such as *smo* (regulating Hedgehog signaling) [44], *fst* (regulating Transforming Growth Factor beta signaling) [45], and *lfng* (regulating Notch signaling) [46] as novel elongation targets of Spt5.

### Analysis of RNAPII occupancy on putative Spt5 target genes in 24 hpf *fog^sk8^* embryos

**Figure 7:**
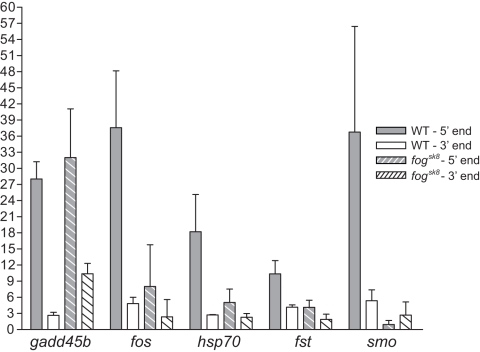
RNAPII occupancy on direct target genes of Spt5 in *fog^sk8^* mutant embryos reveals unexpected complexity and increased 3′ occupancy at the *gadd45b* locus. ChIP with RNAPII antibody in WT and *fog^sk8^* embryos on *gadd45b*, *fos*, *hsp70*, *fst*, and *smo* genes show that in *fog^sk8^* embryos, RNAPII occupancy is significantly increased at 3′ end of *gadd45b* without increase at 5′ end. Other genes tested showed reduction of RNAPII occupancy at 5′ ends but no change at 3′ ends in *fog^sk8^* embryos compared to WT. The results shown are the average of at least three independent replicates. Error bars are SEM values.

Based on the above results and current model of transcription elongation, one might expect, in the *fog^sk8^* embryos, an increased occupancy of RNAPII at 3′ end of the genes whose transcription elongation is repressed by Spt5 in WT embryos. Alternatively, depletion of Spt5 may lead to a decreased association of RNAPII with the 5′ end of genes, due to a reduced level of promoter-proximal pausing, as has been observed in the case of NELF depletion [8]. To test these predictions, *in vivo* ChIP was carried out using RNAPII antibody in 24 hpf *fog^sk8^* embryos. Due to limited availability of *fog^sk8^* embryos, we focused on five genes, *hsp70*, *fos*, *gadd45b*, *fst*, and *smo*. The patterns of RNAPII occupancy in 24 hpf *fog^sk8^* embryos were shown in Fig. 7. For most genes tested, we observed significantly decreased RNAPII occupancy at 5′ end and no increase of occupancy at 3′ end, in comparison to that in WT embryos, consistent with the second prediction. One gene, *gadd45b*, however, fitted with the first prediction: RNAPII occupancy at its 5′ end was similar between WT and *fog^sk8^* mutant embryos, but RNAPII occupancy at its 3′ end was significantly increased in *fog^sk8^* embryos as compared to WT (Fig. 7). Reasons behind such differential patterns of RNAPII occupancy at different target genes were not clear (see Discussion). Taken together, our *in vivo* expression profiling and ChIP analyses indicate that Spt5 exerts dual activity in regulating mRNA expression and RNAPII occupancy *in vivo* and in a gene-specific manner.

### *gadd45b* mRNA is globally increased leading to increased protein products in *fog^sk8^* embryos

**Figure 8:**
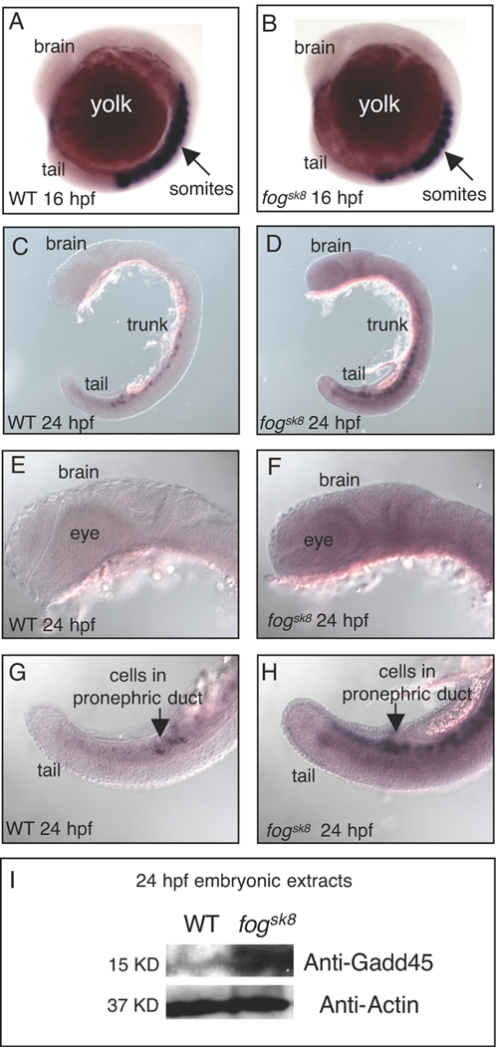
Global increase of *gadd45* mRNA and increased Gadd45b protein in *fog^sk8^* mutant embryos. (A–H) Whole-mount in situ hybridization using the *gadd45b* antisense probe in WT and *fog^sk8^* embryos at 16 hpf and 24 hpf. *gadd45b* expression is not increased at 16 hpf but shows widespread increase at 24 hpf in *fog^sk8^* embryos. (I) Western blot analysis of WT and *fog^sk8^* embryo extracts with commercially available anti-Gadd45b antibody and anti-Actin antibody shows significant increase of Gadd45b protein in *fog^sk8^* embryos at 24 hpf.

The one gene that did show increased RNAPII occupancy in the *fog^sk8^* mutant, *gadd45b*, is regulated by multiple important cellular signaling pathways including NF-kB and JNK [47] and is involved in zebrafish somitogenesis [48]. To probe the significance of Spt5 regulation of this gene, we asked whether and where in the *fog^sk8^* embryos the de-repression of *gadd45b* results in overall mRNA increase, through whole-mount *in situ* hybridization. In 16 hpf WT embryos, *gadd45b* was strongly expressed in the developing somites, and such expression was largely normal in the *fog^sk8^* mutant (Fig. 8A–B). In 24 hpf WT embryos, *gadd45b* expression was detected in cells of pronephric ducts and weakly in posterior paraxial mesoderm and minimally elsewhere (Fig. 8C, 8E, 8G), revealing the dynamic nature of *gadd45b* expression in development. In 24 hpf *fog^sk8^* embryos, a global increase of *gadd45b* RNA (Fig. 8D, 8F, 8H), and protein (Fig. 8I) was detected, thus demonstrating the significance of Spt5 regulation of this gene.

## Discussion

Spt5 has a demonstrated dual activity in regulating transcription elongation *in vitro* [21], and may regulate a large number of genes *in vivo* [28], [29], [49]. However, the prevalence and identity of genes whose expression is critically dependent on Spt5 are poorly defined in any *in vivo* system. In this study, we have carried out expression profiling of over 10,000 genes using a zebrafish zygotic null mutant, *fog^sk8^*. Maternally deposited *foggy/spt5* gene products have sustained *fog^sk8^* embryos to a developmental stage when sufficient amount of tissues are experimentally available and *foggy/spt5* gene products become significantly depleted. Our results show that a surprisingly small fraction of genes (<5%) are differentially expressed between WT and *fog^sk8^* embryos at 24 hpf. *In vivo* ChIP analyses with both Spt5 and RNAPII antibodies on a select set of genes further identify targets that are regulated either by the repressive or by the stimulatory activity of Spt5 at the level of transcription elongation. The observation that Spt5 associates with control genes confirm that Spt5 occupancy does not equate to functional necessity. Moreover, RNAPII occupancy patterns observed in *fog^sk8^* embryos emphasize the dynamic and complex nature of transcription regulation by Spt5 in a gene-specific manner *in vivo*.

### The prevalence of target genes that require Spt5 regulation in vertebrate development

Previous immunolocalization studies using Drosophila polytene chromosomes show that non-productively elongating RNAPII, together with Spt5, labels many chromosomal sites [28], [29]. Recent genome-wide analyses also find significant RNAPII occupancy at the 5′ end of many genes, whether or not their transcripts can be detected [5]–[7]. These studies imply that most, if not all, genes may be dependent on Spt5 for transcription regulation. In contrast to this view, our survey of over 10,000 genes at one developmental stage (24 hpf) reveals less than 5% of differentially expressed genes in *fog^sk8^* embryos. Several possibilities may explain why such a small fraction of genes show altered expression in *fog^sk8^* embryos: First, as localization does not always reflect functional dependence, it is possible that, although a much larger number of genes are bound by Spt5, only a fraction of them are critically dependent on its activity. Indeed, our ChIP analyses have found that Spt5 are associated with two genes whose expression is largely unchanged in *fog^sk8^* embryos. Second, despite significant reduction of Spt5 protein level in *fog^sk8^* embryos, some maternal proteins still remain, which could mask the identification of those genes whose expression is not particularly sensitive to Spt5 protein reduction. Although removal of maternal Spt5 will allow the test of this hypothesis, it may potentially lead to early death of embryos, rendering the experiments impossible to carry out. Third, since this expression profiling experiment cannot distinguish the relative contribution of maternal versus zygotic mRNA to the steady state mRNA levels, it remains possible that maternal mRNAs that are not yet degraded by 24 hpf could mask changes of Spt5-dependent zygotic gene expression. Finally, since only one developmental stage is examined in this study, it remains possible that a different set of genes and/or a much larger number of them may show altered expression in the mutant at other developmental stages. Interestingly, when we analyzed the expression of 21 candidate target genes in a hypomorphic mutation (the *fog^m806^* allele) at 48 hpf, 15 of the genes tested were unchanged while four (*fos*, *ndrg1*, *atp1a1a.4* and *pvalb*) showed similar trend as in *fog^sk8^* mutant (Supplemental Table S5). This observation suggests that the *fog^m806^* allele is likely to affect even smaller number of genes. Taken together, we suggest that, at the late segmentation stage of zebrafish development, only a small fraction of genes is critically dependent on Spt5 for transcription regulation, even though a large number of genes may recruit Spt5 to their chromatin.

### Target genes that are positively or negatively regulated by Spt5

In addition to previously known categories of inducible genes, our pathway analysis revealed that Spt5 target genes belong to diverse biological pathways and functions. Further studies are required to bridge the gap between the known function of the genes to the mechanism of their gene regulation at the elongation level, particularly to developmentally regulated genes. Our bioinformatics analyses reveal that genes repressed by Spt5 are significantly shorter in gene length. Two possibilities, not mutually exclusive, may account for this observation: First, as one distinct advantage of regulating transcription elongation is to allow rapid gene induction in response to extrinsic signals, genes that are regulated in this manner may have been selected during evolution to have shorter length, in order to further accommodate the need for accelerated transcription. Interestingly, genes with shorter introns and overall shorter gene length are hallmarks of immediate early (IE) genes. Since only three of the genes up-regulated in the *fog^sk8^* appear to be well- characterized IE genes (*gadd45b*, *fos* and *jun*), further studies are required to determine if other genes represented in this category also function as IE genes. Second, since the severe depletion of Spt5 in *fog^sk8^* embryos lead to deficits of not only the repressive but also the stimulatory activity, it is possible that the genes with longer length are not up-regulated in *fog^sk8^* embryos because of their dependency on both the repressive as well as the stimulatory activity of Spt5.

Our expression profiling together with *in vivo* ChIP analyses at one developmental stage have identified two non-overlapping sets of target genes: those that are negatively regulated by Spt5 and those that are positively regulated by Spt5. Are there indeed two distinct populations of genes that differentially require Spt5 activity *in vivo*? We suggest not. First, this study found that *hsp70* belongs to the group that is negatively regulated by Spt5. Under heat stimulus, however, it has been previously shown that *hsp70* induction also depends on Spt5 [32]. Thus, one gene may require both the repressive and the stimulatory activity of Spt5, depending on developmental states or surrounding environmental signals. Spt5 is shown to be phosphorylated by the positive elongation kinase P-TEFb [14], [34], [36]–[38], and such phosphorylation is proposed to act as a switch that converts Spt5 from a repressor to an activator [34]. Although such model remains to be tested *in vivo*, it supports the idea that Spt5 and transcription elongation are dynamically regulated *in vivo*, and same set of genes may be regulated by the repressive and stimulatory activity of *foggy/Stp5*, depending on the *in vivo* states.

### Dynamics and complexity of regulated transcription elongation *in vivo*

Our ChIP analyses in *fog^sk8^* embryos reveal decreased RNAPII occupancy along the chromatin of four genes (*fos*, *hsp70*, *fst*, and *smo*) among total five genes tested. Since the transcripts of these genes are increased in *fog^sk8^* embryos at 24 hpf, together with the demonstrated RNAPII and Spt5 occupancy on these genes in WT embryos, one possible explanation is that increased 3′ RNAPII occupancy may have occurred at an earlier developmental stage, coinciding with the onset of transcript increase from these genes. Because of Spt5 depletion, promoter-proximal association of RNAPII was reduced subsequently in *fog^sk8^* mutant embryos, resulting in observed decrease of RNAPII occupancy at 24 hpf. Indeed, it has been shown in yeast that Spt4-5 complex is important for stabilizing RNAPII on the chromatin template under normal and DNA-damage inducing conditions [50]. Similar reduction of promoter-proximal associated RNAPII has also been observed after NELF knockdown in Drosophila [8].

Among all the up-regulated genes subjected to ChIP analysis, *gadd45b* showed increased RNAPII occupancy at the 3′ end in the *fog^sk8^* mutant embryos. It also exhibited highest Spt5 occupancy at the 5′ end in WT (Figure 5A). In addition, the onset of *gadd45b* mRNA increase in *fog^sk8^* embryos may be rather close to 24 hpf, since no increase in *gadd45b* mRNA was detected in 16 hpf *fog^sk8^* embryos. These observations may explain why *gadd45b* displays increased RNAPII occupancy patterns in *fog^sk8^* embryos. The significantly up-regulated Gadd45b protein levels suggest that the produced *gadd45b* transcripts can be translated *in vivo*. In zebrafish, overexpression of *gadd45b* by mRNA injection at the 1-cell stage leads to somite segmentation defect and a down-regulation of *myoD* [48]. Since *gadd45b* was not misexpressed in *fog^sk8^* embryos until late segmentation stages, *fog^sk8^* embryos do not show obvious segmentation defects and no reduction in *myoD* transcript levels was observed in our expression profiling analysis. Thus, understanding the contribution of overexpressed *gadd45b* or any other target genes to the *fog^sk8^* mutant phenotype would require a precise determination of temporal profiles of their expression in the *fog^sk8^* mutant, followed by conditional knockdown or overexpression of these target genes with temporal control.

How might the control of *gadd45b* expression by Spt5 fit into the known regulation of *gadd45b? Gadd45b* is regulated by NF-kappaB [51] and TGF-beta [52] in mammalian cell cultures, though its *in vivo* regulation is poorly understood. Future studies are required to elucidate any potential interaction between Spt5 and the NF-kB and/or TGF-beta pathway members to understand the interplay between DNA binding transcription factors and elongation regulation of *gadd45b*. It has been previously suggested that *gadd45* falls into the category of ‘preset’ genes whose upregulation is independent of changes in chromatin structure (by binding of transcription factors), or synthesis of new transcription factors, or stimuli-induced DNA-protein interactions [53]. Hence, it is tempting to speculate that the elongation control of *gadd45b* by Foggy/Spt5 is the switch that regulates these ‘preset’ genes. Transcription elongation control of such ‘preset’ genes might be crucial to integrate signals from multiple pathways (*gadd45b* controlled by NF-kB and TGF-b) to coordinate regulation of important signaling pathways that ultimately determine cell fate and survival.

Taken together, our findings provide the first comprehensive view of *in vivo* target genes whose transcription is critically dependent on Spt5 both positively and negatively during vertebrate development. Further studies are required to elucidate the complexities of elongation regulation through Spt5 *in vivo*, as well as to determine the significance of such regulation on individual target genes and their associated pathways.

## Materials and Methods

### Zebrafish strains and husbandry

Wild-type AB and *fog^sk8^* embryos were obtained through natural spawning and were maintained and staged according to established procedures [54]. The use of zebrafish was approved by UCSF ethics committee.

### Total RNA isolation and quantitative RT-PCR

Equal number of embryos (WT siblings vs. *fog^sk8^* mutants) at 24 hpf was dechorionated manually or by Pronase K treatment. Trizol (Invitrogen) was used to extract total RNA that was DNase-treated (Qiagen) to minimize contamination by genomic DNA during qRT-PCR. To obtain cDNA, total RNA was reverse-transcribed using oligodT and Superscript (Invitrogen). Equal amount of cDNA was used in SybrGreen qPCR mastermix system (Applied Biosystems) with appropriate primers (Supplemental Table S2). All plates contained at least two-well duplicates for each primer and cDNA condition. Cycle values were averaged between the duplicates and normalized to control primer values. WT fold values were compared to *fog^sk8^* mutant fold values and expressed as fold upregulated or downregulated in *fog^sk8^* embryos. At least two independent replicates were performed for each primer set.

### Microarray Data Analysis

Microarray hybridization was performed at the NINDS microarray consortium using zebrafish Affymetrix oligonucleotide arrays. Three independent replicates for each sample were performed. Gene intensity values for the Affymetrix Zebrafish Genome Array CEL files were obtained using the GC-RMA package from Bioconductor [55]. To identify genes with relative changes in steady-state mRNA levels between WT and *fog^sk8^* mutants, a fold change was calculated from these log2 intensity values along with a student *t test p* value. Genes with 2 fold change in expression and *p*<0.05 were clustered using the program HOPACH (hierarchical ordered partitioning and collapsing hybrid), with uncentered correlation distance [56], [57]. Associations with Gene Ontology, GenMAPP [58] and PANTHER [59]pathways were obtained using MAPPFinder 2.0 [42], a part of the GenMAPP 2.1 [41] application package and further filtered and annotated using the program GO-Elite (http://www.genmapp.org/go_elite/go_elite.html). The raw image and processed expression of our microarray dataset is posted at NCBI's Gene Expression Omnibus database (GSE12826). To determine relative differences in gene length parameters, a custom python script was written that links microarray probe set data to gene structural information from the Ensembl database (PMID: 17148474) obtained through BioMart [60]. Probe set annotations to Ensembl genes obtained from BioMart were augmented with those directly from Affymetrix (http://www.affymetrix.com). In this script, fold changes were calculated by comparing the mean length of distinct gene features (genomic, exon and intron lengths/number) or gene expression between upregulated or downregulated Ensembl-linked genes compared to all genes on the microarray. For each parameter, a permutation *p* value was calculated by randomizing the selection of input probe sets 10,000 times and re-calculating and storing the mean fold changes for both up and down regulated sets to determine the probability that the original fold occurs by chance alone.

### Chromatin Immunoprecipitation (ChIP) Assay

WT embryos at 24 hpf were dechorionated by Pronase treatment and washed thoroughly. Embryos were fixed in 1.5% formaldehyde for 20 minutes and then homogenized 25 times in 5× extraction buffer with protease inhibitors on ice. Sonication was carried out with Branson sonifier 250 on an ice bath for 10 times, 10 seconds each with 20 second breaks. Sonicated samples were then centrifuged at 14k rpm at 4°C for 30 minutes to separate the pellet from the supernatant. The supernatant was immunoprecipitated with the following antibodies: 8WG16 Ab-1∶40 (Covance Research Products), Anti-Spt5 antibody 1∶10, and control sera -Normal mouse serum-1∶100 (Sigma) and Rabbit pre-immune serum (1∶10) for 2–4 hours in the cold room. Protein A Sepharose (Sigma) beads were added to the samples and left on overnight. The next day, extensive washing with different buffers were performed before eluting the beads at 65°C for 20 minutes. Proteinase K and RNase were added to the eluates and reverse-crosslinked overnight at 65°C. Phenol/chloroform extraction followed by ethanol precipitation was performed. The immunoprecipitated DNA was immediately used for qPCRs with appropriate primers (Supplemental Table S4). All cycle values were first normalized to input values before comparing control samples to immunoprecipitated samples. These values were represented as fold occupancy over control values. The procedure was performed similarly for *fog^sk8^* embryos.

### Whole mount in situ hybridization and immunocytochemistry

Whole-mount *in situ* hybridization was performed as previously described [61]. Plasmid containing *pBS-gadd45b* was obtained from ZIRC, and antisense probe was made using T7 RNA polymerase (Ambion). Whole-mount immunocytochemistry was performed similarly with appropriate antibodies (8WG16 ab- 1∶100; Anti-zfSpt5 antibody – 1∶5000; CTD4H8 –AF488 – 1∶2500) and fluorescent secondary antibodies. Images were taken with Zeiss epi-fluorescent and confocal microscopes.

### Western Blot

Dechorionated embryos were fixed in 1.5% formaldehyde for 20 minutes and homogenized before addition of 2× SDS loading buffer. Equal amounts of the extracts were loaded on to the wells of the gel. Primary antibodies (Anti-Foggy antibody-1∶6000; Anti-Actin antibody – 1∶2000; Anti-Gadd45b antibody – 1∶100) and secondary antibody conjugated to horse-radish peroxidase (HRP) were used. The ECL kit (Amersham) was used to visualize the protein bands transferred from the blot to film.

## Supporting Information

Figure S1 (0.88 MB DOC)

Table S1 (0.03 MB DOC)

Table S2 (0.04 MB DOC)

Table S3 (0.08 MB DOC)

Table S4 (0.05 MB DOC)

Table S5 (0.04 MB DOC)
