# Supplementary material for: Identification of Spt5 Target Genes in Zebrafish Development Reveals Its Dual Activity In Vivo
Source: PLoS One. 2008 Nov 3;3(11):e3621. doi: 10.1371/journal.pone.0003621 (PMC2575381; doi:10.1371/journal.pone.0003621)
Supplement: Figure S1 — (0.88 MB DOC) [file pone.0003621.s001.doc]

**
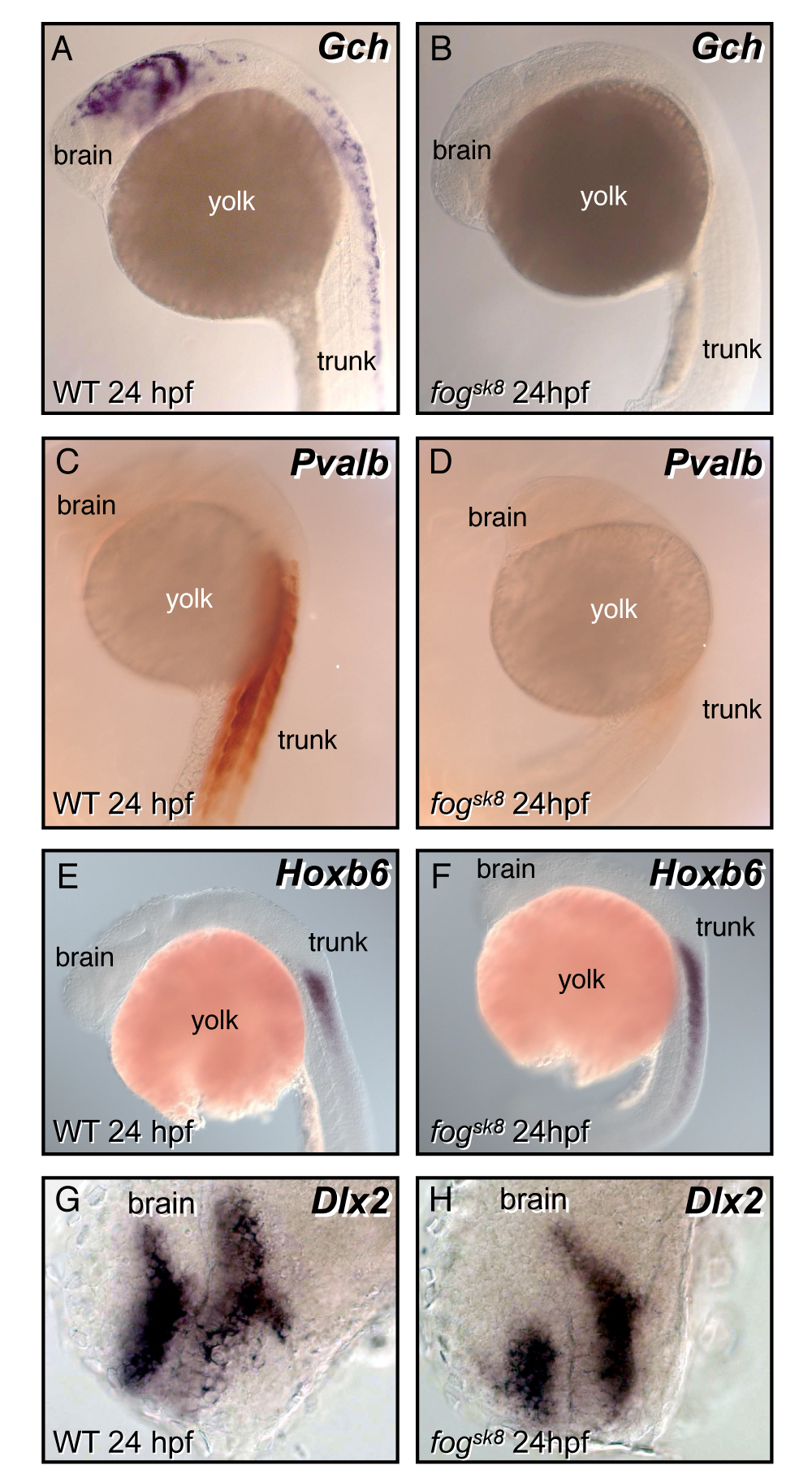
**

**Supplemental Figure 1.** Whole mount in situ hybridization validation of candidate genes in WT (A, C, E, G) and *fog^sk8^* (B, D, F, H) embryos at 24hpf. gch (A, B) and pvalb (C, D) are not expressed (357 fold and 17 fold down-regulation in the array) in fog sk8 embryos. hoxB6 (E, F) expression is up-regulated 2.2 fold while dlx2 (G, H) expression is not changed in *fog^sk8^* embryos.
