# Supplementary material for: Identification of Spt5 Target Genes in Zebrafish Development Reveals Its Dual Activity In Vivo
Source: PLoS One. 2008 Nov 3;3(11):e3621. doi: 10.1371/journal.pone.0003621 (PMC2575381; doi:10.1371/journal.pone.0003621)
Supplement: Table S1 — (0.03 MB DOC) [file pone.0003621.s002.doc]

| **Supplementary Table 1 Gene Expression Validation by Quantitative RT-PCR** | | | | |
| --- | --- | --- | --- | --- |
| **Genes upregulated in f*og^sk8^* embryos** | | |  |  |
| **#** | **Array Probe ID** | Genes | **QRT-PCR folds** | **Array folds** |
| 1 | Dr.592.1.S1_at | foxd5 (Forkhead box D5) | 10.77 | 9.83 |
| 2 | Dr.15991. 1.S1_at | Hypothetical protein | 4.40 | 6.56 |
| 3 | Dr.1378.2.S1_a_at | gadd45b (Growth arrest and DNA-damage-inducible gene, beta) | 6.07 | 6.34 |
| 4 | Dr.12986.2.S1_at | fos (v-fos viral oncogene) | 8.44 | 5.68 |
| 5 | Dr.20198.2.S1_x_at | hsp70 (heat shock protein 70) | 2.32 | 5.60 |
| 6 | Dr.21063.1.A1_at | bapx (NK3 homeobox 2) | 4.62 | 4.62 |
| 7 | Dr.15033.1.S1_at | Hypothetical protein | 7.33 | 4.00 |
| 8 | Dr.13076.1.S1_at | plekhf1 (Pleckstrin homology domain containing, family F member 1) | 3.19 | 3.87 |
| 9 | Dr.23439.4.S1_at | zp2.4 (Zona pellucida glycoprotein 2.4) | 6.02 | 3.74 |
| 10 | Dr.5725.1.S1_at | hoxB6b (homeo box B6b) | 3.84 | 3.31 |
| 11 | Dr.25206.1.S1_at | tpbgl (Trophoblast glycoprotein-like) | 2.37 | 3.24 |
| 12 | Dr.18282.6.A1_at | khdrbs1 (KH domain containing, RNA binding, signal transduction associated 1) | 5.94 | 2.97 |
| 13 | Dr.8097.1.S1_at | opn1sw2 (Opsin 1, short-wave-sensitive 2) | 7.81 | 2.72 |
| 14 | Dr.12334.1.A1_at | Hypothetical protein | 4.24 | 2.35 |
| 15 | Dr.198.1.S1_at | fst (Follistatin) | 2.07 | 2.25 |
| 16 | Dr.15833.1.A1_at | rpb1 (RNA Polymerase II) | 3.71 | 2.17 |
| 17 | Dr.24766.1.S1_at | smo (smoothened homolog) | 2.12 | 2.02 |
| 18 | Dr.14282.1.S1_at | atf3 (activating transcription factor 3) | 1.89 | 2.00 |
| 19 | Dr.24669.1.S1_at | gtf2A (General transcription factor IIA) | 3.61 | 1.82 |
| **Genes downregulated in f*og^sk8^* embryos** | | |  |  |
| 1 | Dr.14668.1.S1_at | gch1 (GTP cyclohydrolase I) | 899.50 | 320.56 |
| 2 | Dr.20928.1.S1_at | pvalb1d (Parvalbumin 1d) | 21.47 | 22.35 |
| 3 | Dr.23350.1.S1_at | pvalb8 (Parvalbumin 8) | 12.23 | 17.62 |
| 4 | DrAffx.1.52.S1_at | a2bp1l (ataxin 2 binding protein 1-like) | 7.83 | 10.69 |
| 5 | Dr.12107.1.A1_at | ndrg1 (N-myc downstream regulated gene 1) | 8.47 | 6.04 |
| 6 | Dr.11483.1.S1_at | ldb3l (LIM domain binding 3-like) | 3.44 | 3.64 |
| 7 | Dr.10719.1.S1_at | atp1a1a.4 (ATPase, Na+/K+ transporting, alpha 1a.4) | 62.85 | 3.37 |
| 8 | Dr.1831.1.S1_at | lfng (Lunatic fringe homolog) | 3.68 | 3.00 |
| 9 | Dr.20815.1.S1_at | tpma (Alpha tropomyosin) | 3.41 | 2.86 |
|  |  |  |  |  |
| **Control genes** | | | | |
| 1 | Dr.25213.1.S1_at | bactin1 (Beta actin 1) | -1.80 | 1.26 |
| 2 | Dr.1347.1.S1_at | rpl4 (Ribosomal protein L4) | 1.00 | 1.00 |
| 3 | Dr.2675.1.A1_at | fkbp5 (FK506 binding protein 5) | 1.00 | 1.04 |
| 4 | Dr.29.1.S1_at | ccne (Cyclin E) | 1.87 | 1.03 |
