# Supplementary material for: Identification of Spt5 Target Genes in Zebrafish Development Reveals Its Dual Activity In Vivo
Source: PLoS One. 2008 Nov 3;3(11):e3621. doi: 10.1371/journal.pone.0003621 (PMC2575381; doi:10.1371/journal.pone.0003621)
Supplement: Table S2 — (0.04 MB DOC) [file pone.0003621.s003.doc]

| **Supplementary Table 2 Primers for Quantitative RT-PCR** | | | | |
| --- | --- | --- | --- | --- |
| Genes up-regulated in *fog^sk8^* embryos | |  |  |  |
| **#** | **Probe ID** | **Genes** | **Forward Primer** | **Reverse Primer** |
| 1 | Dr.592.1.S1_at | foxd5 | GGTGCTCATTCAGCATTGACAGCA | ACCATCCGAAGAGTCTCCGAGAAA |
| 2 | Dr.15991. 1.S1_at | Hypothetical protein | TGACCGACTCTAATGCTGTCCTCA | TGCTGTTGCTGTGTTTATGGGCTG |
| 3 | Dr.1378.2.S1_a_at | gadd45b | TAGCGGAGGAACAAGTTGTGGGAA | ATGTTCGTCCCGCATTCAGCGATA |
| 4 | Dr.12986.2.S1_at | fos | AAACGACGTCTTCCCGACATCAGT | TGTGGCAGGCATGTATGGTTCAGA |
| 5 | Dr.20198.2.S1_x_at | hsp70 | CCTGGAGTCTTACGCCTTCAACAT | CCCTGGTAGAGTTTGGAGATGACT |
| 6 | Dr.21063.1.A1_at | bapx | TCTGTCGCTGCAGCCCTCTTATTA | GGCGTGTTCGGTAAACGAAAGTCT |
| 7 | Dr.15033.1.S1_at | Hypothetical protein | GAGGGAAAGGCAGGAGTAAGAAGG | GGACCACAATCCGGTTTCTTTCGTC |
| 8 | Dr.13076.1.S1_at | plekhf1 | ACCTGTGAGGGTTTGCCGAAATTG | AGTACGCAGAGAAACTGTCCTCCT |
| 9 | Dr.23439.4.S1_at | zp2.4 | AGTGTGCTACCCTTCAGCTACTCA | CACCACGGCAGTGTCATCAGAAAT |
| 10 | Dr.5725.1.S1_at | hoxb6b | AACAGGCATCGAACTCACCTCTGT | AACACACGTGGACTGAATACAGGA |
| 11 | Dr.25206.1.S1_at | tpbgl | AAGAAGCGGATTTACGACATGCGG | ACATCGGCTGTTGAAGACACTTGC |
| 12 | Dr.18282.6.A1_at | khdrbs1 | TGAGCATTAGCACGCAGTTTCCTG | ACTGTCTGAACCCAAGAGTAGTT |
| 13 | Dr.8097.1.S1_at | opn1sw2 | TAACCTCATTCTGCGGACGAACCA | CATGTTCAGCAAGCCAAGACCAAG |
| 14 | Dr.12334.1.A1_at | Hypothetical protein | GTCAATGGTCACCTGTTTCCAGCA | GCGTCAGGCATATCCAAATGTTGC |
| 15 | Dr.198.1.S1_at | fst | ACAACACCACGTATCCCAGTGAGT | TTGCTGGGCAGCATTGGATTGTCT |
| 16 | Dr.15833.1.A1_at | rpb1 | ATGCTTGAACAGTAGAGGGCGACA | AGCTGCTCTCCCTAATGCAGTCTA |
| 17 | Dr.24766.1.S1_at | smo | AGAATTCAGGCCTTCGTGTCCACT | TTAGGTTGCCATGAGCTTTGCTGG |
| 18 | Dr.14282.1.S1_at | atf3 | CAGCCCTGTGACTGCATTGCCTAATA | GGAAACAAACAAACTAGATGACGCCTGC |
| 19 | Dr.24669.1.S1_at | tfIIa | CGTGGTTCTGTGCATTGTGGAGTT | CTGAAAGGCACAAACAAGGAGC |
| Genes down-regulated in *fog^sk8^* embryos | | |  |  |
| 1 | Dr.14668.1.S1_at | gch1 | TCGCAGACTTCAAGTTCAAGAGCG | TAAGGGATGGAAGTGTGATCACTGCG |
| 2 | Dr.20928.1.S1_at | pval1d | ACACCTCGACTAGCTCCTTTGCTT | GCACATGCCAGTGGGCTTGAAATA |
| 3 | Dr.23350.1.S1_at | pvalb8 | AACTCATCCGTCTGGACACTCGAT | CATGTGCGCTCTCTACATGTCCAA |
| 4 | DrAffx.1.52.S1_at | a2bp1l | ACCGCCACATACAGTGATGGGTAA | AACTCCATAAGTGGTTGTGGGTCC |
| 5 | Dr.12107.1.A1_at | ndrg1 | ACCAATCAGTTCTGACTGTGCTGC | TGGCGTAAATGCTCTCCGACTACT |
| 6 | Dr.11483.1.S1_at | ldb3k | GCCTGTCTGCACAGATCCTAAACA | TGATTCCTGGTTGAATGCTGCCTC |
| 7 | Dr.10719.1.S1_at | atp1a1a.4 | CAGGCATGGACATTGCAGTCAGAA | CTGGATTCCGTCGCAGGATGTATT |
| 8 | Dr.1831.1.S1_at | lfng | TGCCTCGGTATCTGAAAGGCATGA | CACCAGGCAGGAAGGAACTGTTTA |
| 9 | Dr.20815.1.S1_at | tpma | GCTCCTTCTCATCACCTCATGTTG | CCTGCACTGTGGATTCCTATGGTA |
| Control genes | |  |  |  |
| 1 | Dr.25213.1.S1_at | bactin1 | TGGCATTGCTGACCGTATGCAGAA | AGCACTTCCTGTGAACGATGGATG |
| 2 | Dr.1347.1.S1_at | rpl4 | CATTCGTCCTGCAAACCGCAAGAT | AGCCTTGGTCTTCTTCACAACCCT |
| 3 | Dr.2675.1.A1_at | fkbp5 | TAAGCCGCTACGCACAGGTTGTAT | ATGAATGCCAGTCGGAGAGCTGAA |
| 4 | Dr.29.1.S1_at | ccne | TATCCGCTAGTGGACATTGAGAGC | AAGAGATGGTGCTCTCTGGCTTCT |
