# Supplementary material for: Identification of Spt5 Target Genes in Zebrafish Development Reveals Its Dual Activity In Vivo
Source: PLoS One. 2008 Nov 3;3(11):e3621. doi: 10.1371/journal.pone.0003621 (PMC2575381; doi:10.1371/journal.pone.0003621)
Supplement: Table S3 — (0.08 MB DOC) [file pone.0003621.s004.doc]

| **Supplemental Table 3 Analysis of FGF, WNT, and BMP pathway genes** | | | | | | |
| --- | --- | --- | --- | --- | --- | --- |
| **#** | **Gene ID** | **Gene Symbol^a^** | | **Gene Title** | **f*og^sk8^* –WT fold** | **mean expression^b^ (1-14)** |
| FGF Pathway genes | | | | | | |
| 1 | Dr.10434.1.S1_at | fgfr3 | | fibroblast growth factor receptor 3 | -1.38 | 7.90 |
| 2 | Dr.10640.1.S1_at | fgf4 | | fibroblast growth factor 4 | 1.07 | 2.08 |
| 3 | Dr.12614.1.S1_a_at | fgfr1 | | fibroblast growth factor receptor 1 | -1.00 | 2.19 |
| 4 | Dr.17781.1.A1_at | fgf20a | | fibroblast growth factor 20a | 1.05 | 3.31 |
| 5 | Dr.19222.1.S1_at | fgfr2 | | fibroblast growth factor receptor 2 | -1.38 | 4.95 |
| 6 | Dr.19222.1.S2_at | fgfr2 | | fibroblast growth factor receptor 2 | -1.15 | 7.59 |
| 7 | Dr.20979.1.S1_at | fgf17a | | fibroblast growth factor 17 a | 1.00 | 2.73 |
| 8 | Dr.24029.1.S1_at | fgfr1op2 | | FGFR1 oncogene partner 2 | 1.49 | 6.93 |
| 9 | Dr.25473.1.A1_at | fgf18 | | fibroblast growth factor 18 | 1.04 | 2.16 |
| 10 | Dr.26455.1.S1_at | fgfrl1a | | fibroblast growth factor receptor-like 1a | -1.37 | 6.28 |
| 11 | Dr.409.1.S1_at | fgfr4 | | fibroblast growth factor receptor 4 | -1.16 | 8.55 |
| 12 | Dr.478.1.S1_at | fgf8 | | fibroblast growth factor 8 | 1.12 | 4.60 |
| 13 | Dr.5775.1.S1_at | fgf3 | | fibroblast growth factor 3 | -1.05 | 2.66 |
| 14 | Dr.8853.1.S1_at | fgf10 | | fibroblast growth factor 10 | -1.50 | 4.86 |
| 15 | DrAffx.1.59.S1_at | fgf2 | | fibroblast growth factor 2 | 1.02 | 1.98 |
| 16 | DrAffx.1.75.S1_at | fgf24 | | fibroblast growth factor 24 | -1.01 | 2.38 |
| 17 | DrAffx.1.76.S1_at | fgf6 | | fibroblast growth factor 6 | 1.04 | 2.31 |
| **WNT Pathway genes** | | | | | | |
| 1 | DrAffx.1.19.S1_at | bsk146 | | brain specific kinase 146 | 1.05 | 2.68 |
| 2 | DrAffx.1.13.S1_at | ankrd6 | | ankyrin repeat domain 6 | -1.01 | 2.13 |
| 3 | Dr.8297.1.S1_at | wnt11r | | wingless-type MMTV integration site family, member 11, related | -1.45 | 7.13 |
| 4 | Dr.8294.1.S1_at | axin1 | | axin 1 | -1.14 | 5.40 |
| 5 | Dr.8292.1.S1_at | tcf7l1a | | transcription factor 7-like 1a (T-cell specific, HMG-box) | -1.27 | 4.70 |
| 6 | Dr.8236.1.S1_at | wnt4b | | wingless-type MMTV integration site family, member 4b | -1.01 | 2.16 |
| 7 | Dr.8108.1.S1_at | tcf7l1b | | transcription factor 7-like 1b (T-cell specific, HMG-box) | -1.09 | 6.27 |
| 8 | Dr.8056.1.S1_at | dkk1 | | dickkopf 1 | -1.13 | 4.92 |
| 9 | Dr.7824.1.S1_at | rhoab | | ras homolog gene family, member Ab | 1.08 | 9.27 |
| 10 | Dr.623.1.S1_at | wnt8b | | wingless-type MMTV integration site family, member 8b | 1.01 | 1.99 |
| 11 | Dr.6107.1.S1_at | myst2 | | MYST histone acetyltransferase 2 | -1.06 | 7.96 |
| 12 | Dr.605.1.S1_at | wnt11 | | wingless-type MMTV integration site family, member 11 | 1.26 | 4.47 |
| 13 | Dr.5398.1.A1_at | dvl2 | | dishevelled, dsh homolog 2 (Drosophila) | 1.02 | 6.13 |
| 14 | Dr.5365.1.A1_at | dusp5 | | dual specificity phosphatase 5 /// similar to Dual specificity phosphatase 5 | -1.03 | 3.90 |
| 15 | Dr.4007.1.S1_at | sp5l | | Sp5 transcription factor-like | 1.41 | 3.77 |
| 16 | Dr.389.1.S1_at | wnt5b | | wingless-type MMTV integration site family, member 5b | -1.16 | 2.74 |
| 17 | Dr.385.1.S1_at | wnt4a | | wingless-type MMTV integration site family, member 4a | -1.23 | 2.19 |
| 18 | Dr.378.1.S1_at | wnt2 | | wingless-type MMTV integration site family member 2 /// similar to Wnt2 | -1.01 | 2.93 |
| 19 | Dr.3727.1.S1_a_at | axin2 | | axin 2 (conductin, axil) | -1.72 | 8.40 |
| 20 | Dr.3690.1.S1_at | wif1 | | wnt inhibitory factor 1 | 1.05 | 5.41 |
| 21 | Dr.342.1.S1_at | wnt10a | | similar to Wnt10a protein | 1.03 | 2.23 |
| 22 | Dr.3137.1.A1_at | dvl2 | | Dishevelled, dsh homolog 2 (Drosophila) | 1.19 | 5.38 |
| 23 | Dr.309.1.S1_at | wnt8a | | wingless-type MMTV integration site family, member 8a /// wnt8-like protein 2 | 1.05 | 1.82 |
| 24 | Dr.2966.1.A1_at | myst1 | | MYST histone acetyltransferase 1 | -1.06 | 7.94 |
| 25 | Dr.25291.3.A1_at | ppp1cb | | protein phosphatase 1, catalytic subunit, beta isoform | -1.02 | 9.21 |
| 26 | Dr.24985.1.S1_at | prickle1 | | prickle-like 1 (Drosophila) | 1.27 | 3.39 |
| 27 | Dr.24894.1.S1_at | wnt10b | | wingless-type MMTV integration site family, member 10b | -1.07 | 2.14 |
| 28 | Dr.2413.1.S1_at | dusp1 | | dual specificity phosphatase 1 /// hypothetical protein LOC554991 | 1.05 | 9.39 |
| 29 | Dr.2395.1.A1_at | ublcp1 | | ubiquitin-like domain containing CTD phosphatase 1 | -1.14 | 6.57 |
| 30 | Dr.20561.1.A1_at | klhl12 | | kelch-like 12 (Drosophila) /// hypothetical protein LOC554584 | 1.58 | 6.71 |
| 31 | Dr.19933.1.S1_at | wnt2b | | wingless-type MMTV integration site family, member 2B | 1.02 | 2.03 |
| 32 | Dr.17859.1.S1_at | ublcp1 | | ubiquitin-like domain containing CTD phosphatase 1 | 1.13 | 5.68 |
| 33 | Dr.17733.1.S1_at | axin1 | | axin 1 | 1.04 | 6.88 |
| 34 | Dr.16519.1.S1_at | metap2 | | methionyl aminopeptidase 2 | -1.12 | 9.51 |
| 35 | Dr.16331.1.A1_at | mapk3 | | Mitogen-activated protein kinase 3 | 1.14 | 8.33 |
| 36 | Dr.16331.2.S1_at | mapk3 | | Mitogen-activated protein kinase 3 | 1.30 | 6.53 |
| 37 | Dr.16301.1.S1_at | dusp6 | | Dual specificity phosphatase 6 | 1.29 | 2.77 |
| 38 | Dr.16301.2.A1_at | dusp6 | | dual specificity phosphatase 6 /// similar to dual specificity phosphatase 6 isoform b | 1.12 | 8.43 |
| 39 | Dr.15701.1.S1_at | wnt1 | | wingless-type MMTV integration site family, member 1 | 1.01 | 1.49 |
| 40 | Dr.13169.1.S1_at | ripk5 | | receptor interacting protein kinase 5 | -1.28 | 7.35 |
| 41 | Dr.12506.1.S1_at | ck2b | | casein kinase 2 beta | 1.08 | 10.04 |
| 42 | Dr.12148.1.S1_at | invs | | inversin | 1.03 | 2.21 |
| 43 | Dr.11996.1.A1_at | LOC569991 | | similar to wnt inhibitory factor 1 | -1.91 | 4.13 |
| 44 | Dr.11481.1.A1_at | zgc:92153 | | zgc:92153 | 1.06 | 2.44 |
| 45 | Dr.11043.1.A1_at | dixdc1 | | DIX domain containing 1 | -1.58 | 5.74 |
| 46 | Dr.10697.1.S1_at | dvl3 | | dishevelled, dsh homolog 3 (Drosophila) | -1.01 | 6.38 |
| BMP Pathway Genes | | | | | | |
| 1 | Dr.8289.1.S1_at | | bmpr1b | bone morphogenetic protein receptor, type 1b | -1.18 | 5.38 |
| 2 | Dr.8154.1.S1_at | | bmpr1a | bone morphogenetic protein receptor, type 1a | -1.03 | 6.83 |
| 3 | Dr.8129.1.S1_at | | smad1 | MAD homolog 1 (Drosophila) | -1.35 | 5.76 |
| 4 | Dr.8123.1.S1_at | | nog2 | noggin 2 | 1.08 | 3.46 |
| 5 | Dr.8120.1.S1_at | | nog1 | noggin 1 | -1.20 | 3.16 |
| 6 | Dr.8119.1.S1_at | | nog3 | noggin 3 | -1.02 | 2.47 |
| 7 | Dr.8110.1.S1_at | | bmp7 | bone morphogenetic protein 7 /// hypothetical protein LOC554381 | 1.11 | 2.58 |
| 8 | Dr.7989.1.A1_at | | bmpr1ab | bone morphogenetic protein receptor, type IA,b | -1.35 | 6.61 |
| 9 | Dr.7919.1.S1_at | | bambi | BMP and activin membrane-bound inhibitor (Xenopus laevis) homolog | 1.09 | 7.93 |
| 10 | Dr.7003.1.A1_at | | bmp15 | bone morphogenetic protein 15 | 1.04 | 2.15 |
| 11 | Dr.7003.2.S1_at | | bmp15 | bone morphogenetic protein 15 | 1.02 | 2.02 |
| 12 | Dr.606.1.S2_at | | acvr1 | ACVR1 activin A receptor, type I | 1.24 | 7.80 |
| 13 | Dr.571.1.S1_at | | tll1 | tolloid-like 1 | 1.02 | 1.98 |
| 14 | Dr.568.1.S1_at | | bmp2b | bone morphogenetic protein 2b /// similar to bone morphogenetic protein 2 /// similar to bone morphogenetic protein 2 | 1.68 | 5.31 |
| 15 | Dr.567.2.S1_a_at | | bmp4 | bone morphogenetic protein 4 /// hypothetical protein LOC555125 | 1.02 | 5.40 |
| 16 | Dr.26149.1.A1_at | | smad5 | MAD homolog 5 (Drosophila) | 1.05 | 2.64 |
| 17 | Dr.25497.1.S1_at | | szl | sizzled | 1.04 | 2.39 |
| 18 | Dr.23348.1.A1_at | | bmp3 | bone morphogenetic protein 3 | 1.27 | 6.71 |
| 19 | Dr.20558.1.S1_at | | smad5 | MAD homolog 5 (Drosophila) | -1.07 | 7.80 |
| 20 | Dr.16432.1.A1_at | | gdf6a | growth differentiation factor 6a | 1.33 | 5.92 |
| 21 | Dr.15550.1.A1_at | | LOC558505 | similar to Bmp1 protein | -1.40 | 7.24 |
| 22 | Dr.15495.1.A1_at | | bmp6 | bone morphogenetic protein 6 | 1.87 | 3.20 |
| 23 | Dr.1463.1.S1_at | | bmp2a | bone morphogenetic protein 2a | 1.28 | 3.56 |
| 24 | Dr.13384.1.S1_at | | chd | chordin | 1.04 | 2.58 |
| 25 | Dr.12376.1.A1_at | | bmper | BMP binding endothelial regulator | 1.10 | 6.81 |
| 26 | Dr.11728.1.S1_at | | twsg1b | twisted gastrulation homolog 1b (Drosophila) | -1.02 | 4.04 |
| 27 | Dr.10625.1.A1_at | | bmp5 | bone morphogenetic protein 5 | -2.24 | 7.49 |

a. Genes that are associated with the FGF, BMP, Wnt signaling pathways were identified through GO terms and/or gene symbol and collected in this table.

Most of these genes are not differentially expressed in *fog^sk8^* embryos at 24 hpf.

b. The mean expression level of genes in WT is categoized from 1-14, with 1 being lowest, and 14 being highest.
