# Supplementary material for: Identification of Spt5 Target Genes in Zebrafish Development Reveals Its Dual Activity In Vivo
Source: PLoS One. 2008 Nov 3;3(11):e3621. doi: 10.1371/journal.pone.0003621 (PMC2575381; doi:10.1371/journal.pone.0003621)
Supplement: Table S4 — (0.05 MB DOC) [file pone.0003621.s005.doc]

| **Supplementary Table 4. Primers for ChIP analysis** | | | | |
| --- | --- | --- | --- | --- |
| **#** | **ProbeID Number** | **Gene Symbol** | **5' - Forward primer** | **5' - Reverse Primer** |
| 1 | Dr.1378.2.S1_a_at | gadd45b | CGAGCAAAGGAATACTGCAACTCC | ATCCAACGACTTCTTCCAGGGTCA |
| 2 | Dr.12986.2.S1_at | fos | AGAGCAGCAGACGAGCAAGGAAAT | CTGAGTCTGGTTGAGCGGGTAATA |
| 3 | Dr.20198.2.S1_x_at | hsp70 | CCAGCATAGACTTCGCGATAGAAC | GTCAGGCGTATTGATGGCTTGTTG |
| 4 | Dr.198.1.S1_at | fst | CAGCACTGCCTGAGTAAGAAGA | GCTGACGCTTTAGCATCCTTAGCA |
| 5 | Dr.24766.1.S1_at | smo | AGTGGATCCATGGTCTTCATCCGA | TCTGGGACTGGGACTGTAACTTGA |
| 6 | Dr.10032.1.S1_at | atf3 | AGCCGGCGACAACTGTAACTTTAG | ATGCCGTCTCTTACCTGGTTGTGT |
| 7 | Dr.25206.1.S1_at | tpbgl | AGTGAAGTTAAGAGGGAGGAGCAC | GTCCACGGGCAAACATGGTGAAAT |
| 8 | Dr.592.1.S1_at | foxd5 | ATGACCCTCTCGCAGGATTACGAA | AGGCTTGACGGAGGAGCTTTGTTT |
| 9 | Dr.21063.1.A1_at | bapx | TCGATGGACCGCGTAATCTTAACC | CACGCGCTCTTTGAGAAACACACA |
| 10 | Dr.23439.4.S1_at | zp2.4 | GTTTCTGGGACTTTGTGCCTGGTT | CACTTTGCTGCACCGTCAATCCAT |
| 11 | Dr.8097.1.S1_at | opn1sw2 | TGAGAGGGAGGTGACAAAGATGGT | ACGGGATTGTACACTGTAGAGGCT |
| 12 | Dr.1831.1.S1_at | lfng | AGCAGTCGGATGACACGCTTGTTT | CTGCGAGAGAAACAACAGAAACCC |
| 13 | Dr.20815.1.S1_at | tpma | GAAGCCTCTCTCAGTCCAGTGTTT | TCGAGCTTGAGCATCTGCATCTTC |
| 14 | DrAffx.1.52.S1_at | a2bp1l | TCCTACTGTGATCCTTCAGCCCTA | TTTACCTGAACAATGCCGGG |
| 15 | Dr.12107.1.A1_at | ndrg1 | CCGGACATTAAACGGGTAAGTCGT | AGCTATCTTCCTCGATATCACGTC |
| 16 | Dr.11483.1.S1_at | ldb3l | ATCCCGACTGGAGTGAGAACAACT | GGAGATGGTCAGGGACATGTTGAA |
| 17 | Dr.460.1.A1_at | pvalb8 | TTAGTTAGGTGGGTAATCTGAGCGCC | TCCGGCAGGTGAATGAACAGAAGA |
| 18 | Dr.10719.1.S1_at | atp1a1a.4 | TTTGCATGGCCAGACAGACACTTG | CCAACCAGTTCACCAGCATCCAAA |
| 19 | Dr.25213.1.S1_at | bactin1 | ACAGCCATGGATGAGGAAATCGCT | TCTCCATGTCATCCCAGTTGGTCA |
| 20 | Dr.29.1.S1_at | ccne | ATGCTGCCTGCTTACAGACTACTG | TCCAAGGGTATCCTCGACTGCAAA |
| 21 | Dr.2675.1.A1_at | fkbp5 | CCCACGTTCACAAACACACTGCTT | GCACAGCAGCATCAGTGAATACCA |

| **#** | **ProbeID Number** | **Gene Symbol** | **3'- Forward Primer** | **3' - Reverse Primer** |
| --- | --- | --- | --- | --- |
| 1 | Dr.1378.2.S1_a_at | gadd45b | TAGCGGAGGAACAAGTTGTGGGAA | ATGTTCGTCCCGCATTCAGCGATA |
| 2 | Dr.12986.2.S1_at | fos | AAACGACGTCTTCCCGACATCAGT | TGTGGCAGGCATGTATGGTTCAGA |
| 3 | Dr.20198.2.S1_x_at | hsp70 | CCTGGAGTCTTACGCCTTCAACAT | CCC TGG TAG AGT TTG GAG ATG ACT |
| 4 | Dr.198.1.S1_at | fst | ACAACACCACGTATCCCAGTGAGT | TTGCTGGGCAGCATTGGATTGTCT |
| 5 | Dr.24766.1.S1_at | smo | AGAATTCAGGCCTTCGTGTCCACT | TTAGGTTGCCATGAGCTTTGCTGG |
| 6 | Dr.10032.1.S1_at | atf3 | CAGCCCTGTGACTGCATTGCCTAATA | GGAAACAAACAAACTAGATGACGCCTGC |
| 7 | Dr.25206.1.S1_at | tpbgl | AAGAAGCGGATTTACGACATGCGG | ACATCGGCTGTTGAAGACACTTGC |
| 8 | Dr.592.1.S1_at | foxd5 | GGTGCTCATTCAGCATTGACAGCA | ACCATCCGAAGAGTCTCCGAGAAA |
| 9 | Dr.21063.1.A1_at | bapx | TCTGTCGCTGCAGCCCTCTTATTA | GGCGTGTTCGGTAAACGAAAGTCT |
| 10 | Dr.23439.4.S1_at | zp2.4 | AGTGTGCTACCCTTCAGCTACTCA | CACCACGGCAGTGTCATCAGAAAT |
| 11 | Dr.8097.1.S1_at | opn1sw2 | TAACCTCATTCTGCGGACGAACCA | CATGTTCAGCAAGCCAAGACCAAG |
| 12 | Dr.1831.1.S1_at | lfng | TGCCTCGGTATCTGAAAGGCATGA | CACCAGGCAGGAAGGAACTGTTTA |
| 13 | Dr.20815.1.S1_at | tpma | GCTCCTTCTCATCACCTCATGTTG | CCTGCACTGTGGATTCCTATGGTA |
| 14 | DrAffx.1.52.S1_at | a2bp1l | ACCGCCACATACAGTGATGGGTAA | AACTCCATAAGTGGTTGTGGGTCC |
| 15 | Dr.12107.1.A1_at | ndrg1 | ACCAATCAGTTCTGACTGTGCTGC | TGGCGTAAATGCTCTCCGACTACT |
| 16 | Dr.11483.1.S1_at | ldb3l | GCCTGTCTGCACAGATCCTAAACA | TGATTCCTGGTTGAATGCTGCCTC |
| 17 | Dr.460.1.A1_at | pvalb8 | AACTCATCCGTCTGGACACTCGAT | CATGTGCGCTCTCTACATGTCCAA |
| 18 | Dr.10719.1.S1_at | atp1a1a.4 | CAGGCATGGACATTGCAGTCAGAA | CTGGATTCCGTCGCAGGATGTATT |
| 19 | Dr.25213.1.S1_at | bactin1 | TGGCATTGCTGACCGTATGCAGAA | AGCACTTCCTGTGAACGATGGATG |
| 20 | Dr.29.1.S1_at | ccne | TATCCGCTAGTGGACATTGAGAGC | AAGAGATGGTGCTCTCTGGCTTCT |
| 21 | Dr.2675.1.A1_at | fkbp5 | TAAGCCGCTACGCACAGGTTGTAT | ATGAATGCCAGTCGGAGAGCTGAA |
