# Supplementary material for: Identification of Spt5 Target Genes in Zebrafish Development Reveals Its Dual Activity In Vivo
Source: PLoS One. 2008 Nov 3;3(11):e3621. doi: 10.1371/journal.pone.0003621 (PMC2575381; doi:10.1371/journal.pone.0003621)
Supplement: Table S5 — (0.04 MB DOC) [file pone.0003621.s006.doc]

| **Supplemental Table 5 Summary of Gene Expression Comparison between *fog* mutants** | | | |
| --- | --- | --- | --- |
| No. | Genes | Array/qRT-PCR Fold Change (*fog sk8*) | qRT-PCR Fold Change (*fog m806*) at 48hpf |
| **Genes Up-regulated in 24 hpf *fog^sk8^*Mutant Embryos** | | |  |
| 1 | *gadd45b* | 6.34/6.07 | 1.76 |
| 2 | *fos* | 5.68/8.44 | 2.36 |
| 3 | *hsp70* | 5.6/2.32 | 1.79 |
| 4 | *fst* | 2.25/2.07 | 1.39 |
| 5 | *smo* | 2.02/2.12 | 1.08 |
| 6 | *atf3* | 2/1.89 | 1.53 |
| 7 | *tpbgl* | 3.24/2.37 | 1.34 |
| 8 | *foxd5* | 9.83/10.77 | 1.57 |
| 9 | *bapx* | 4.62/4.62 | 1.82 |
| 10 | *zp2.4* | 3.74/6.02 | 1.41 |
| 11 | *opn1sw2* | 2.72/7.81 | -2.68 |
| **Genes Down-regulated in 24 hpf *fog^sk8^*Mutant Embryos** | | |  |
| 1 | *lfng* | 3/3.68 | 1.59 |
| 2 | *tpma* | 2.86/3.41 | 1.58 |
| 3 | *a2bp1l* | 10.69/7.83 | 1.18 |
| 4 | *ndrg1* | 6.04/8.47 | 2.41 |
| 5 | *ldb3l* | 3.64/3.44 | 1.22 |
| 6 | *pvalb8* | 17.62/12.23 | 4.44 |
| 7 | *atp1a1a.4* | 3.37/62.85 | 5.49 |
| **Genes Unchanged in 24 hpf *fog^sk8^* Mutant Embryos** | | |  |
| 1 | *bactin1* | 1.26/-1.8 | 1.07 |
| 2 | *ccne* | 1/1.87 | 1.56 |
| 3 | *fkbp5* | 1/1.04 | -4.3 |
